# Proteome Mapping of the Human Pancreatic Islet Microenvironment Reveals Endocrine–Exocrine Signaling Sphere of Influence

**DOI:** 10.1016/j.mcpro.2023.100592

**Published:** 2023-06-15

**Authors:** Sara J.C. Gosline, Marija Veličković, James C. Pino, Le Z. Day, Isaac K. Attah, Adam C. Swensen, Vincent Danna, Camilo Posso, Karin D. Rodland, Jing Chen, Clayton E. Matthews, Martha Campbell-Thompson, Julia Laskin, Kristin Burnum-Johnson, Ying Zhu, Paul D. Piehowski

**Affiliations:** 1 Pacific Northwest National Laboratories, Richland, Washington, USA; 2 Department of Pathology, Immunology and Laboratory Medicine, University of Florida, Gainesville, Florida, USA; 3 Department of Chemistry, Purdue University, West Lafayette, Indiana, USA

**Keywords:** proteomics, networks, mass spectrometry, pancreas, islets, differential expression

## Abstract

The need for a clinically accessible method with the ability to match protein activity within heterogeneous tissues is currently unmet by existing technologies. Our proteomics sample preparation platform, named microPOTS (Microdroplet Processing in One pot for Trace Samples), can be used to measure relative protein abundance in micron-scale samples alongside the spatial location of each measurement, thereby tying biologically interesting proteins and pathways to distinct regions. However, given the smaller pixel/voxel number and amount of tissue measured, standard mass spectrometric analysis pipelines have proven inadequate. Here we describe how existing computational approaches can be adapted to focus on the specific biological questions asked in spatial proteomics experiments. We apply this approach to present an unbiased characterization of the human islet microenvironment comprising the entire complex array of cell types involved while maintaining spatial information and the degree of the islet’s sphere of influence. We identify specific functional activity unique to the pancreatic islet cells and demonstrate how far their signature can be detected in the adjacent tissue. Our results show that we can distinguish pancreatic islet cells from the neighboring exocrine tissue environment, recapitulate known biological functions of islet cells, and identify a spatial gradient in the expression of RNA processing proteins within the islet microenvironment.

## Highlights

• microPOTS enables deep proteome imaging of pancreatic islets and their microenvironment.
• We identified and quantified over 6000 proteins from ∼50,000 μm^2^ tissue area samples.
• Differential expression and network analyses identified pathways uniquely active in islets.
• Rank-based statistics enable the characterization of gradients across individual tissue images.

## Graphical Abstract

**Figure:**
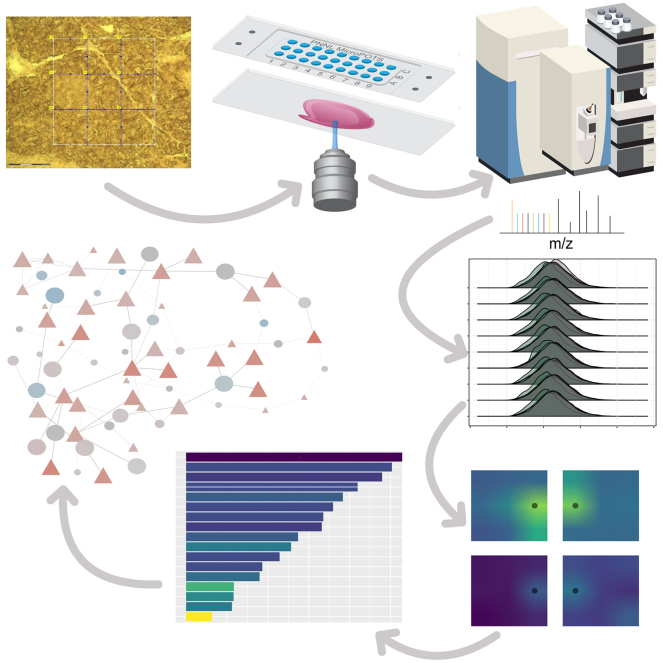

## In Brief

We applied our Microdroplet Processing in One pot for Trace Samples (microPOTS) TMT platform to study the pancreatic islet microenvironment in the human pancreas. We were able to quantify over 6000 proteins from seven different regions within the same patient. Here we describe enhancements to existing computational approaches to extract functional hypotheses from this first-of-its-kind dataset. In addition to recapitulating known functions of the islet, we can identify specific immune and RNA processing activities with spatial heterogeneity in expression.

The islets of Langerhans are endocrine micro-organs embedded within a mostly exocrine pancreas, comprising roughly 2% of the pancreas by mass (1). Islets have been studied for decades, primarily because of their involvement in diseases such as diabetes and obesity (2, 3). Until recently, in-depth protein profiling of pure islets has been very difficult, partly due to their diminutive size and limited compositional make up. Recent cutting-edge technologies have greatly enhanced our understanding of the islet proteome by isolating islets from their surrounding tissues allowing them to be studied down to near single-cell spatial resolution (4, 5, 6). In-depth proteomic studies of acinar cell tissues from the exocrine pancreas have also been demonstrated in the past (7, 8). However, despite their encapsulated nature, islets do not act entirely independently and rely on the surrounding exocrine microenvironment for feedback signaling and crosstalk (9, 10). A characterized islet-acinar portal system directly facilitates islet hormone dispersion in nearby acinar cells. For example, acinar cells are known to contain islet-hormone-specific receptors that regulate acinar function and under the right conditions saturate with locally high concentrations of insulin and somatostatin (11). In addition to insulin and somatostatin, other humoral factors including pancreastatin and ghrelin and several neurotransmitters (nitric oxide, peptide YY, substance P, and galanin) have been shown to be involved in this islet-acinar connection, regulating the functions of each tissue type (12). Through causes we do not yet understand, people with type 1 diabetes and their first-degree relatives have also been shown to have overall reduced pancreatic volume compared to matched controls and some evidence supports exocrine pancreas atrophy and exocrine insufficiency in people with long-term T1D (13, 14, 15). The more we learn about this endocrine–exocrine/islet–acinar connection, the greater its importance appears to be in understanding diseases involving the pancreas. However, until now no unbiased spatially resolved method has been available for deep proteomic investigations of the islet microenvironment encompassing all the cell/tissue types involved this complex system.

Large bulk samples of pancreatic tissues quickly dilute and drown out the contribution of the islet signature. To gain a deeper understanding of the biological signaling and interactions that underlie this endocrine–exocrine connection, it is critical to study the tissues within their original spatial context intact (*i.e.*, *in vivo*) (16, 17). Currently, few technologies are available to study the heterogeneity of biological signaling across a tissue sample. Although there are several powerful techniques for measuring transcripts with high depth and spatial resolution, transcripts often do not correlate well with protein expression (18). Existing technologies for spatially resolved protein measurements mainly rely on the use of tagged antibodies, such as Immunohistochemistry (19), CyTOF (20), and CODEX (21, 22). While these technologies are highly effective and can provide a single-cell level or better spatial resolution, protein coverage is limited by the availability of reliable antibodies and the multiplexing limit of the labels. Imaging mass spectrometry (MALDI, Laser Ablation) is also a powerful tool for protein mapping that does not depend on antibody recognition; but due to the direct coupling to the mass spectrometer, these techniques are limited in their dynamic range and accuracy of quantitation, particularly in clinical samples (23, 24, 25, 26, 27).

Over the last decade, improvements in sensitivity and sample handling for LC-MS proteomics have enabled spatially resolved measurements (28, 29, 30, 31, 32, 33, 34) and have extended to more difficult-to-analyze samples such as formalin-fixed paraffin-embedded (FFPE) tissue (29, 34, 35). More recently, additional advances have enabled single-cell resolution including capillary micro-sampling LC-MS (36), and capillary electrophoreses-based mass spectrometry (37, 38, 39, 40, 41) have enabled the measurement of proteins in very small samples. These approaches are particularly attractive as they offer a comprehensive, quantitative protein profile without *a priori* knowledge of the proteins of interest. In our lab, we have successfully combined laser capture microdissection and the nanoPOTS approach (42, 43), to enable spatially resolved proteome profiling. This initial effort resulted in a platform capable of quantifying >2000 proteins at 100 μm spatial resolution without the use of antibodies or labels (44). To further improve the depth of protein coverage, we next incorporated tandem mass tags (TMT) and nanoflow fractionation and concatenation (nanoFAC (45)) to our proteome profiling workflow. To collect and process enough protein material to facilitate robust fractionation, we scaled up the platform to the microliter scale (46, 47), and incorporated a TMT carrier channel (48, 49) into the image plexes. These changes increased protein coverage to >5000 proteins while maintaining high-quality quantitative information, thus enabling spatially resolved, unbiased interrogation of biological signaling.

Robust computational analysis tools for the analysis of data from these evolving technologies are less established. Technologies such as CyTOF and CODEX have proprietary software packages that are sold with their technology (22), though there are many open-source tools that leverage these data to characterize cells by their protein expression *via* flow cytometry (50, 51, 52). There are also computational packages designed for spatially resolved transcriptomics data that can be leveraged for proteomic analysis, including those that enable mapping and analysis of imaging data (53, 54) as well as those that link the two data modalities to improve protein identification and clustering (55). Existing tools, however, are limited to the study of preformatted image data based on the established platform (*e.g.* Visium (56)) and therefore are not easily applied to microPOTS data.

In this work, we demonstrate the potential utility for microPOTS spatial proteomics to be employed in a clinical setting through the study of multiple pancreas regions within a single patient. While our technical advancements have been described previously (57), here we describe how we enhance existing computational tools to show how these measurements can be used to robustly measure activity across disparate regions within a single pancreas and to make biologically functional hypotheses from these data. In addition to showing the increased insulin signaling activity we know to be present within the islet cells, we also identify specific immune-related processes and RNA processing activities that could not be captured at the transcriptomic level and can be studied further in disease settings such as pancreatic cancer or diabetes.

## Experimental Procedures

### Pancreatic Tissue Section

Human pancreas tissue for microPOTS profiling was obtained from a 17-year-old male donor. The donor was selected based on our eligibility criteria established by the HuBMAP consortium, under IRB201600029 (https://www.protocols.io/view/donor-eligibility-criteria-and-pancreas-recovery-f-b7nfrmbn), and following the ethical standards of the Declaration of Helsinki. Organ recovery and tissue processing were performed at the University of Florida per standard protocol (https://www.protocols.io/view/human-pancreas-processing-b7gxrjxn). To ensure tissue integrity for proteomics measurements ischemia times were tightly controlled during collection with warm ischemia time <60 min and cold ischemia time <18 h, which are sufficient to preserve the global proteome.

### Experimental Design

**Fig. 1:**
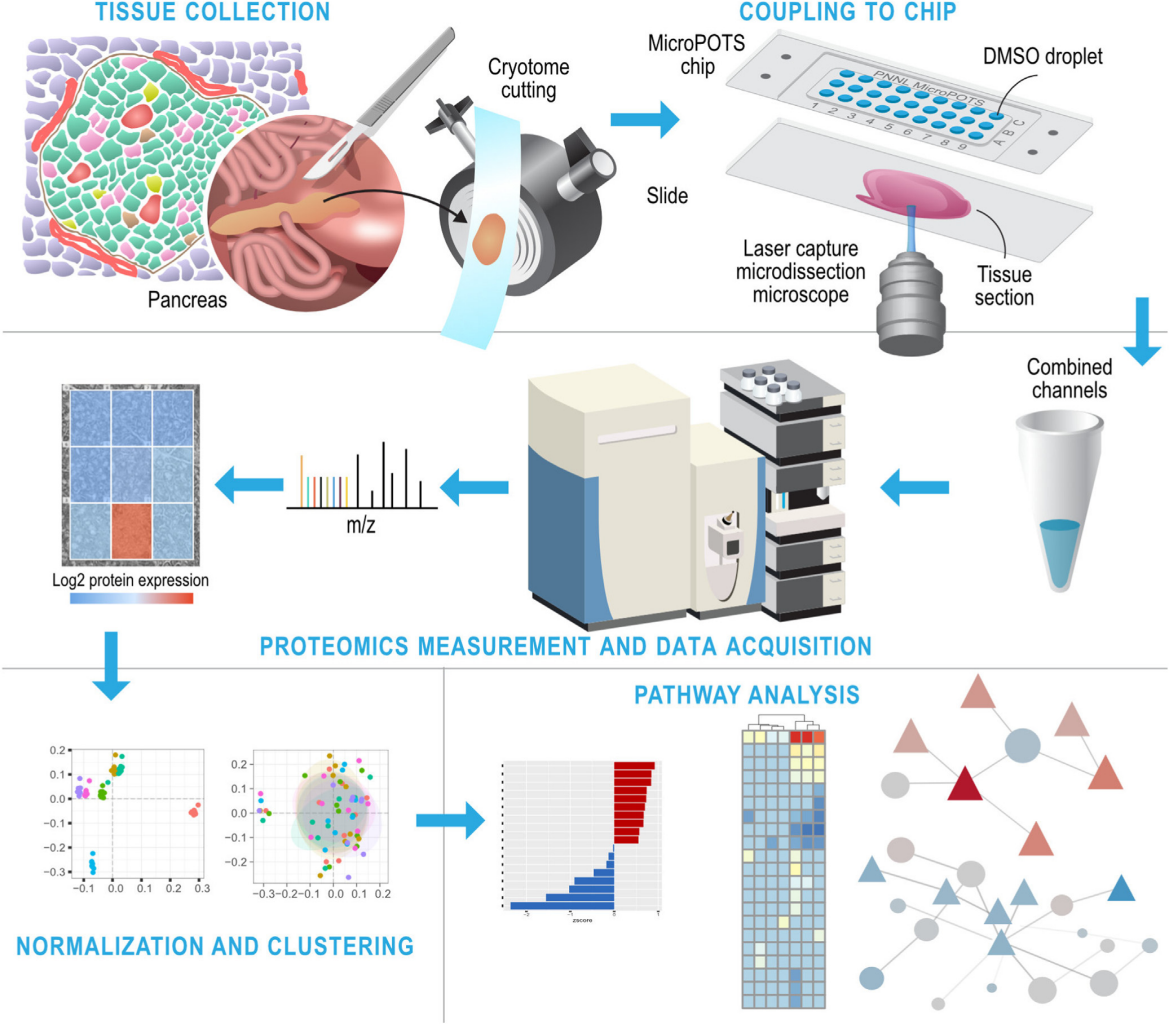
**Overview of our experimental procedures.***top*: Tissue collection and coupling to chip requires laser capture microdissection of flash frozen pancreatic samples enables dissection of each “voxel” into each microwell for sample preparation. *Middle*: each well is individually prepared for TMT labeling and MS/MS. To measure relative protein abundance for each voxel. *Bottom*: Individual voxels are annotated to carry out pathway enrichment and network analysis.

**Supplemental Figure S1:**
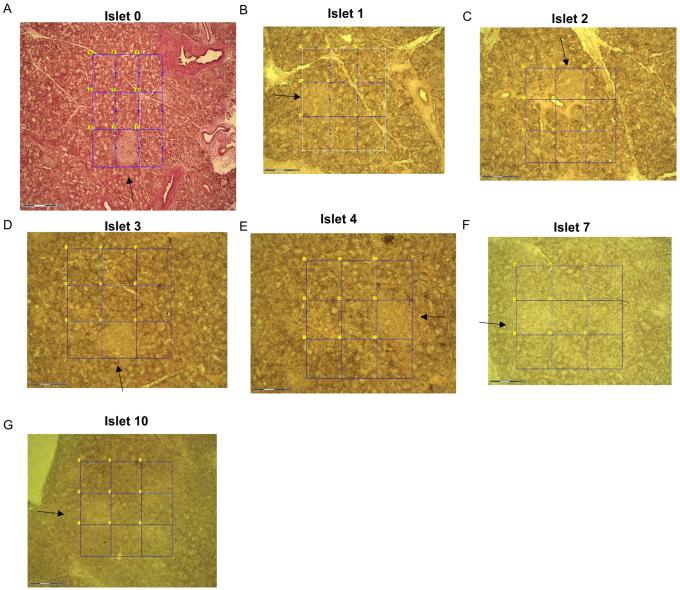
**Description of pancreas sampling and 3 × 3 grid.***A*, image and islet position of image 0. *B*, image and islet position of image 1. *C*, image and islet position of image 2 (*D*) image and islet position of image 3. € image and islet position of image 4. *F*, image and islet position of image 7. *G*, image and islet position of image 10.

Our overall analysis pipeline is depicted in Figure 1 and described below. Seven proteome images were created from seven different regions of a pancreas tissue section taken from a healthy human donor. Images consist of nine tissue “voxels” created by dissecting a 3 × 3 grid from the tissue collected directly into corresponding wells in a microPOTS chip (supplemental Fig. S1). Imaging areas were created from regions containing a singular group of islet cells to interrogate the islets and their unique microenvironment. Grids were sized to capture the islet within a single voxel consisting almost entirely of islet cells. Below we describe how we captured the data to identify islet-specific signaling activity.

### Tissue Collection and Coupling to Chip

#### Tissue Section Preparation

Samples were washed with a gradient of ethanol solutions (70%, 96%, and 100% ethanol, respectively) to dehydrate the tissue sections and to remove embedding material. Briefly, the pancreas was sliced into 0.5-cm-thick tissue segments, subdivided, and immediately frozen in Carboxymethylcellulose (CMC, prepared in Cryotray molds that were prechilled on dry ice/isopentane slurry) (https://www.protocols.io/view/freezing-and-formalin-fixation-of-tissue-br4fm8tn). Frozen CMC tissue blocks were stored at −80 °C until sectioning. CMC embedded human pancreas tissue was cut to 10-μm-thick slices using a cryostat and collected on PEN membrane slides. Serial sections were shipped on dry ice to PNNL for further microPOTS profiling.

#### Laser Capture Microdissection

Sample dissection and “voxel” collection were completed using a PALM Microbeam system (Carl Zeiss MicroImaging) which contains a RoboStage for high-precision laser micromanipulation in the micrometer range and a PALM RoboMover that collects voxel samples directly into the wells of the microPOTS chip. Microwells were preloaded with 3 μl of dimethyl sulfoxide (DMSO) that served as a capturing medium for excised voxels.

For proteomics imaging experiments, we first stained a 10-μm thick adjacent human pancreas section using Periodic Acid-Schiff (PAS) staining kit following the manufacturer’s protocol. The staining for the confident determination of islet and acinar tissue regions when observed using brightfield microscopy. Informed by the islet localization from the serial PAS-stained section, a 3 × 3 grid was created over an islet and the surrounding acinar tissue. The grid was arranged to capture the whole islet in a single pixel of approximately 200 μm × 300 μm dimensions while the surrounding eight pixels contained exclusively acinar tissue. Voxels were dissected using the grid mode and collected directly into corresponding microwells of the chip. A carrier sample was also collected from the same patient tissue section. The carrier sample contained a similar-sized islet and surrounding acinar tissue as the total image area, with a total area equivalent to the entire grid size (∼500,000 μm^2^). This resulted in a 10× carrier to sample ratio.

#### Proteomics Sample Processing in a Microdroplet

All sample handling steps, from extraction through to TMT labeling, were carried out on-chip by manual pipetting. Evaporation during preparation was minimized by cooling during dispensing of reagents, using a humidified chamber for incubation steps, and sealing the chip with a contactless cover and wrapping in aluminum foil. Tissue voxels were incubated at 75 °C for 1 h to remove DMSO solvent. Next, 2 μl of extraction buffer containing 0.1% DDM, 0.5× PBS, 38 mM TEAB, and 1 mM TCEP was dispensed to each well of the chip, followed by incubation at 75 °C for 1 h. We then added 0.5 μl of 10 mM IAA solution in 100 mM TEAB to reach a final concentration of 2 mM IAA followed by incubation at room temperature for 30 min. Samples were subsequently digested by dispensing 0.5 μl of an enzyme mixture (10 ng of Lys-C and 40 ng of trypsin in 100 mM TEAB) and incubated at 37 °C for 10 h. TMT-11 plex reagents were resuspended in anhydrous acetonitrile at a concentration of 6.4 μg/μl. 1 μl of each TMT tag was used to label voxel samples. Following our experimental design, each plex represented a single image, created by leaving the 130N channel empty and using the 131N channel for the carrier sample, 128N channel was used for the islet voxel and the other eight channels for the acinar tissue voxels. The peptide–TMT mixtures were incubated for 1 h at room temperature, and the labeling reaction was quenched by adding 1 μl of 5% HA in 100 mM TEAB and incubating 15 min at room temperature. All samples were then pooled together, brought up to the final 1% FA, then centrifuged at 10,000 rpm for 5 min at 25 °C. Finally, the pooled sample was transferred to an autosampler vial and dried in a speed vac.

#### Reagents and Chemicals

Microwell chips with a 2.2 mm well diameter were manufactured on polypropylene substrates by Protolabs. LC-MS grade water, formic acid (FA), iodoacetamide (IAA), Triethylammonium bicarbonate (TEAB), TMT-10plex and TMT11 to 131C reagents, Anhydrous acetonitrile, Tris(2-carboxyethyl)phosphine hydrochloride (TCEP-HCl), and 50% Hydroxylamine (HA) were all purchased from Thermo Fisher Scientific. N-Dodecyl β-d-maltose (DDM), DMSO (HPLC grade), and Phosphate-Buffered Saline (PBS) and PAS staining kit were purchased from Sigma-Aldrich. Both Lys-C and trypsin were purchased from Promega. Ethanol was purchased from Decon Labs, Inc.

### Proteomic Measurement and Data Acquisition

#### Nanoflow LC-Fractionation

Prior to injection, samples were resuspended in 62 μl of 0.1% formic acid. High pH fractionation was performed offline by loading 50 μl of the sample onto a precolumn (150 μm i.d., 5 cm length) using 0.1% formic acid at a flow rate of 9 μl/min for 9 min. The sample is then pushed onto the LC column (75 μm i.d., 60-cm length) using the separation gradient. Precolumn and column were packed inhouse with 5-μm and 3-μm Jupiter C18 packing material (300-Å pore size) (Phenomenex, Terrence, USA), respectively. An Ultimate 3000 RSLCnano system (Thermo Scientific) was used to deliver gradient flow to the LC column at a nanoflow rate of 300 nl/min. 10 mM ammonium formate (pH 9.5) was used as mobile phase A and acetonitrile as mobile phase B. Eluted fractions were collected using a HTX PAL collect system into autosampler vials preloaded with 25 μl 0.1% formic acid and 0.01% (m/v) DDM. The PAL autosampler allows concatenation on-the-fly by robotically moving the dispensing capillary among 12 collection vials. A total of 96 fractions were concatenated into 12 fractions. Vials were stored at −20 °C until the following low-pH LC-MS/MS analysis.

#### LC-MS/MS Peptide Analyses

LC-MS/MS analysis was carried out using the Ultimate 3000 RSLCnano system (Thermo Scientific), coupled to a Q Exactive HF-X (Thermo Scientific) mass spectrometer. Full MS1 scans were acquired across scan range of 300 to 1800 m/z at a mass resolution of 60,000, combined with a maximum injection time (IT) of 20 ms and automatic gain control (AGC) target value of 3e6. Data dependent MS2 scans were collected using a top 12 method with a resolving power of 45,000, a maximum injection time of 100 ms, and AGC target value of 1e5, with the isolation window was set to 0.7 m/z and dynamic exclusion time was set to 45 s to reduce repeated selection of precursor ions.

#### Data Analysis

Instrument RAW files were first processed using MSConvert to correct mass errors (58). Corrected spectra were searched with MS-GF + v9881 (59, 60) against the Uniprot human database downloaded in March of 2021 (20,371 proteins) and a list of common contaminants (*e.g.*, trypsin, keratin). A partially tryptic search setting was used and a ±20 parts per million (ppm) parent ion mass tolerance. The minimum peptide length was set to 6, and the maximum to 50 amino acids. A reversed sequence decoy database approach was used to control the false discovery rate. Carbamidomethylation (+57.0215 Da) on Cys residues, and TMT modification (+229.1629 Da) on N terminus and Lys residues were considered as static modifications. Oxidation (+15.9949 Da) of Met residues was set as a dynamic modification. Identifications were first filtered to a 1% false discovery rate (FDR) at the unique peptide level, and a sequence coverage minimum of six per 1000 amino acids was used to maintain a 1% FDR at the protein level after assembly by parsimonious inference. Total protein and peptide counts are in supplemental Tables S1 and S2 respectively.

TMT 11 reporter ions area under the curve (AUC) intensities were extracted using MASIC software (61). Extracted intensities were linked to peptide-to-spectrum matches (PSMs) passing the FDR thresholds described above. Intensities were median centered across channels within individual images. Log2 intensities are used for downstream analysis.

#### Image Annotation

We collected seven distinct samples from a single human pancreas and dissected each sample into a 3 × 3 grid creating nine “voxels” for each image (supplemental Fig. S1). Employing our TMT microPOTS pipeline, we were able to quantify 6693 unique proteins across the 63 individual voxels (supplemental Table S1). Each voxel was labeled by its proximity to the known islet cell. As such, one voxel in each image was labeled “Islet,” the five voxels immediately adjacent to the islet were labeled “proximal,” and the three remaining islets were labeled as “distal.”

### Normalization and Statistical Rationale

**Fig. 2:**
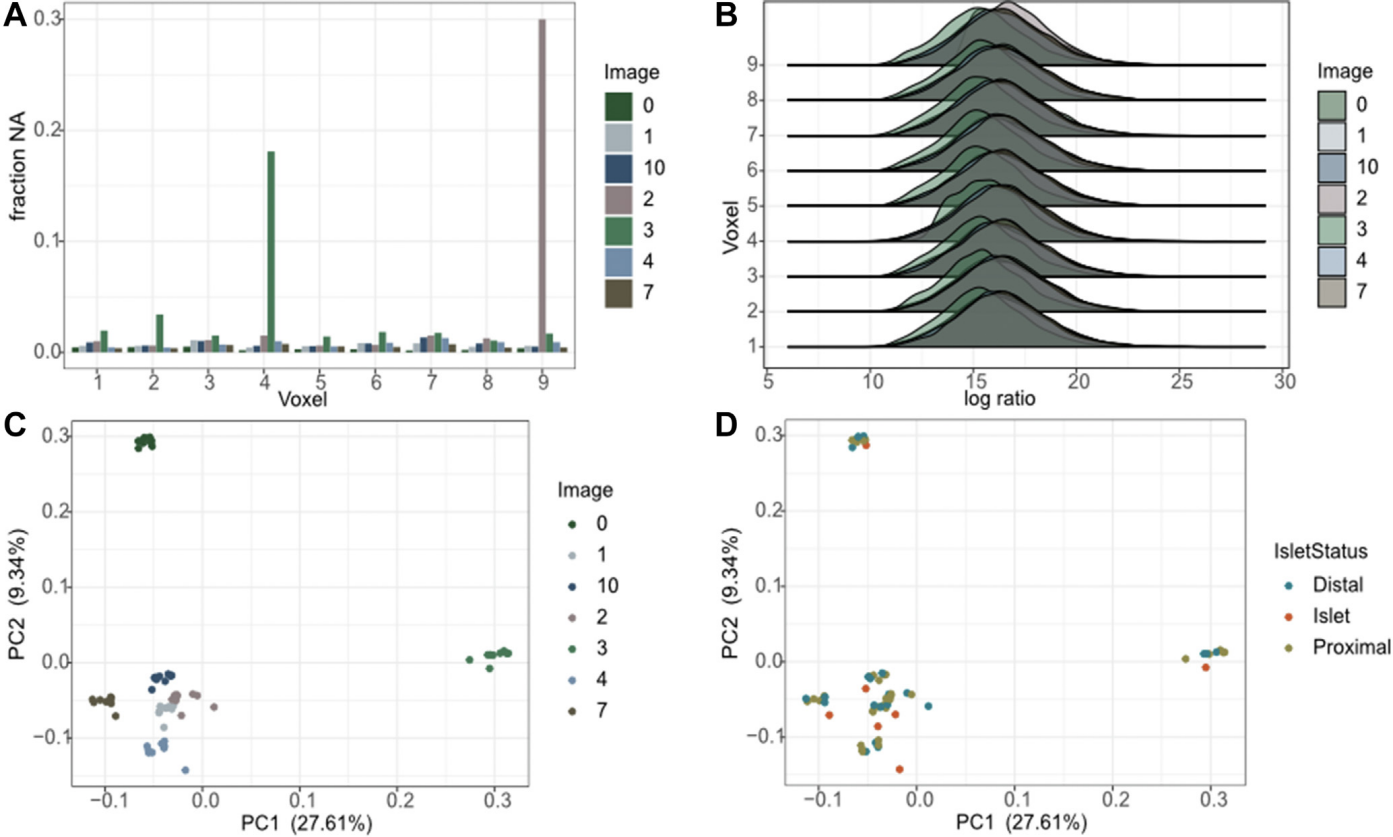
**Protein value distribution.***A*, the fraction of missing values for each voxel (x-axis) for each image. *B*, the distribution of protein log ratio values across each voxel (Y-axis) of each image/plex (color). *C* and *D*, the first two principal components of the complete dataset, colored by image/plex in (*C*) and islet annotation in (*D*).

Each image was analyzed as a separate TMT plex, and therefore subject to batch effects across the 6693 proteins measured. We first looked at missingness in each voxel, depicted in Figure 2*A*. We found that the number of missing values varied with a minimum of nine proteins missing (in Image 0, Grid number 7) and a maximum of 1603 proteins missing (in Image 2, Grid number 9). While Image 2 had the highest number of missing proteins in that single voxel, Image 3 had the highest median number of missing values at 86. We imputed the missing values of each protein with the median expression of that protein across all voxels.

We then considered the need for median centering across plexes, which we often do to account for technical differences between the batches that often confound downstream analysis. We plotted the distribution of log ratio values (Fig. 2*B*) and found that they were not normally distributed as is typically assumed for median centering. Furthermore, we plotted the values in the first two principal components and found that while the samples clustered by image as expected, (Fig. 2*C*), there was still clear separation of the islet regions (Fig. 2*D*). Therefore, we decided against centering the data and decided to include image/plex as a variable in our differential expression calculations going forward.

### Pathway Analysis

To compute differences between the islet containing voxels and other regions, we used the limma package with all seven image plexes pooled with individual voxels annotated as described earlier. In this analysis, we treated both region and image as variables in the analysis, to account for the batch effects noted earlier. This enabled each image to be its own biological replicate, particularly important for the islet cells, which were only measured in one voxel per image. Thus, by using each image as a separate replicate we calculated proteins that were differentially expressed between similarly annotated regions (supplemental Table S2). We then compared analysis of regions using the upsetR (62) tool to compare expression differences across annotated regions, then employed leapR (63) pathway enrichment tool to identify specific pathways that were up regulated in islets across all seven images using the enrichment_in_sets parameter with the KEGG, Reactome, and GO Biological Process pathways. We used the same pathways to do the enrichment_in_order analysis in our variance-based and distance-based analysis. The network analysis leveraged the PCSF R tool (64) based on the approach described previously (65).

## Results

Here we describe the capabilities of the analysis pipeline by which we investigate the proteomics imaging of the microPOTS framework to enable interpretation in biological use cases.

### Spatial Proteomics Across Multiple images Enables Quantitation of Proteins at High Spatial Resolution

As described above, we collected seven distinct image plexes from a single human pancreas and dissected each region into a 3 × 3 grid resulting in 9 “voxels” for each image (supplemental Fig. S1). The samples were then labeled with TMT and fractionated into 12 fractions for subsequent MS analysis quantifying >6000 distinct proteins (supplemental Table S3).

**Fig. 3:**
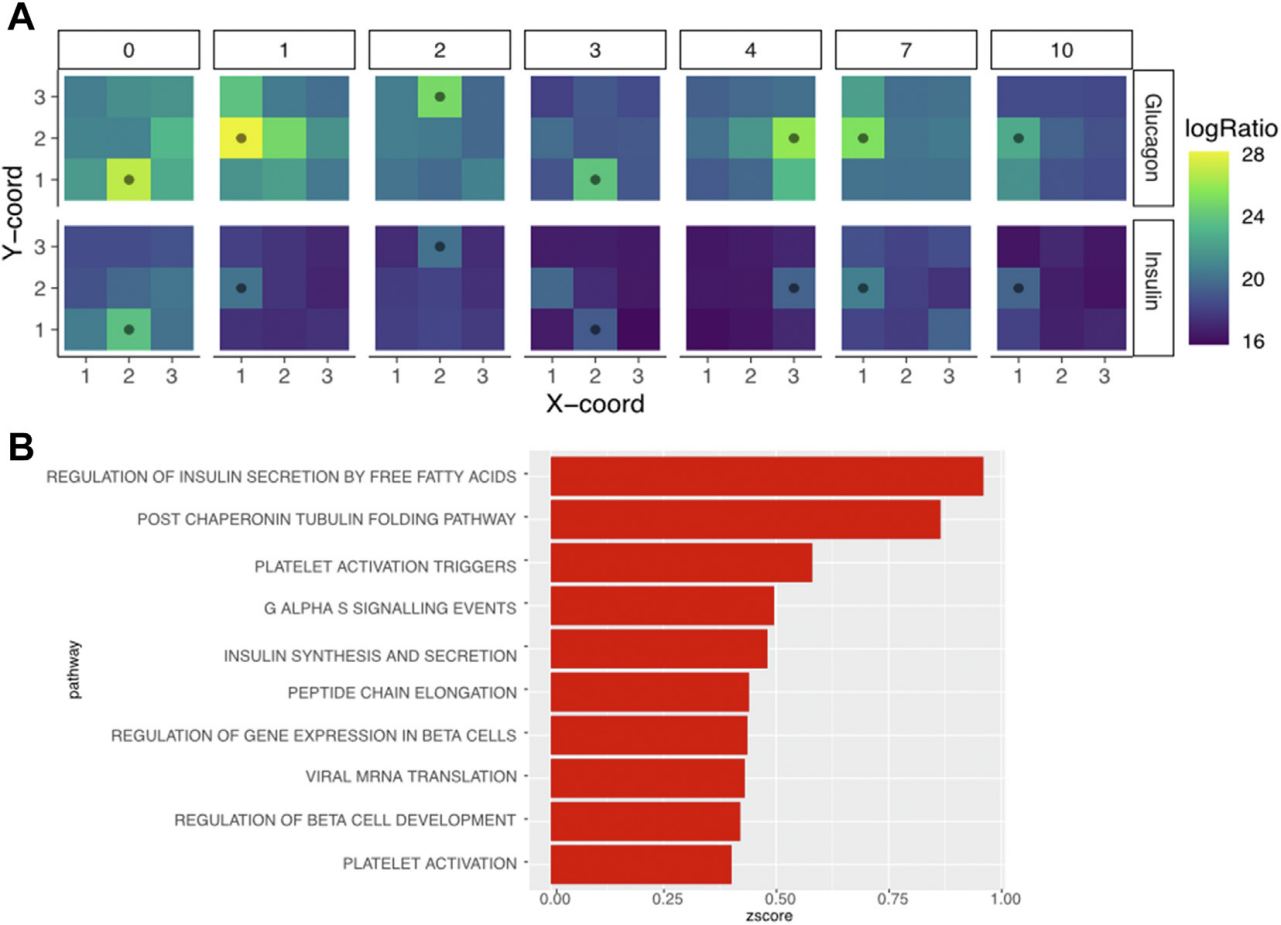
**Assessing protein expression.***A*, confirmation of islet annotation by plotting the log ratio values of known islet-specific proteins including glucagon (*top row*) and insulin (*bottom row*) across all seven images. *Black dot* indicates the voxel that contains the islet. *B*, pathways significantly (corrected *p* < 0.05) enriched when sorting proteins by variance across all voxels show the biological activity most represented by the region.

We first sought to identify proteins that we knew *a priori* have high cell-type specificity. Figure 3*A* shows the uncentered log intensity ratios for glucagon and insulin, hormones released in islet cells, across each of the seven images. Each islet cell is annotated with a dot as derived from the imaging data. As expected, both insulin and glucagon are highest in the islet cells across the seven images. Note that we were unable to resolve beta cells (insulin) from alpha cells (glucagon) at this level of spatial resolution, and that insulin is also highly expressed in a non-islet-containing voxel in image 3, likely due to an islet in another region that was not measured.

Finally, we assess the variance of each protein in all voxels to determine if proteins in some pathways are changing more than others across each of the images. We then used ranked gene set enrichment (see Experimental Procedures) to identify if the variance of proteins corresponded to particular pathways. The results, depicted in Figure 3*B* show that most of the pathways that are highly variable—depicted in red—are related to insulin or beta cells, suggesting that the assay is capturing the expected differences between the beta cells and acinar tissue.

### Image Pooling Increases Replicates to Enable Capture of Islet-Specific Enrichment Patterns

**Fig. 4:**
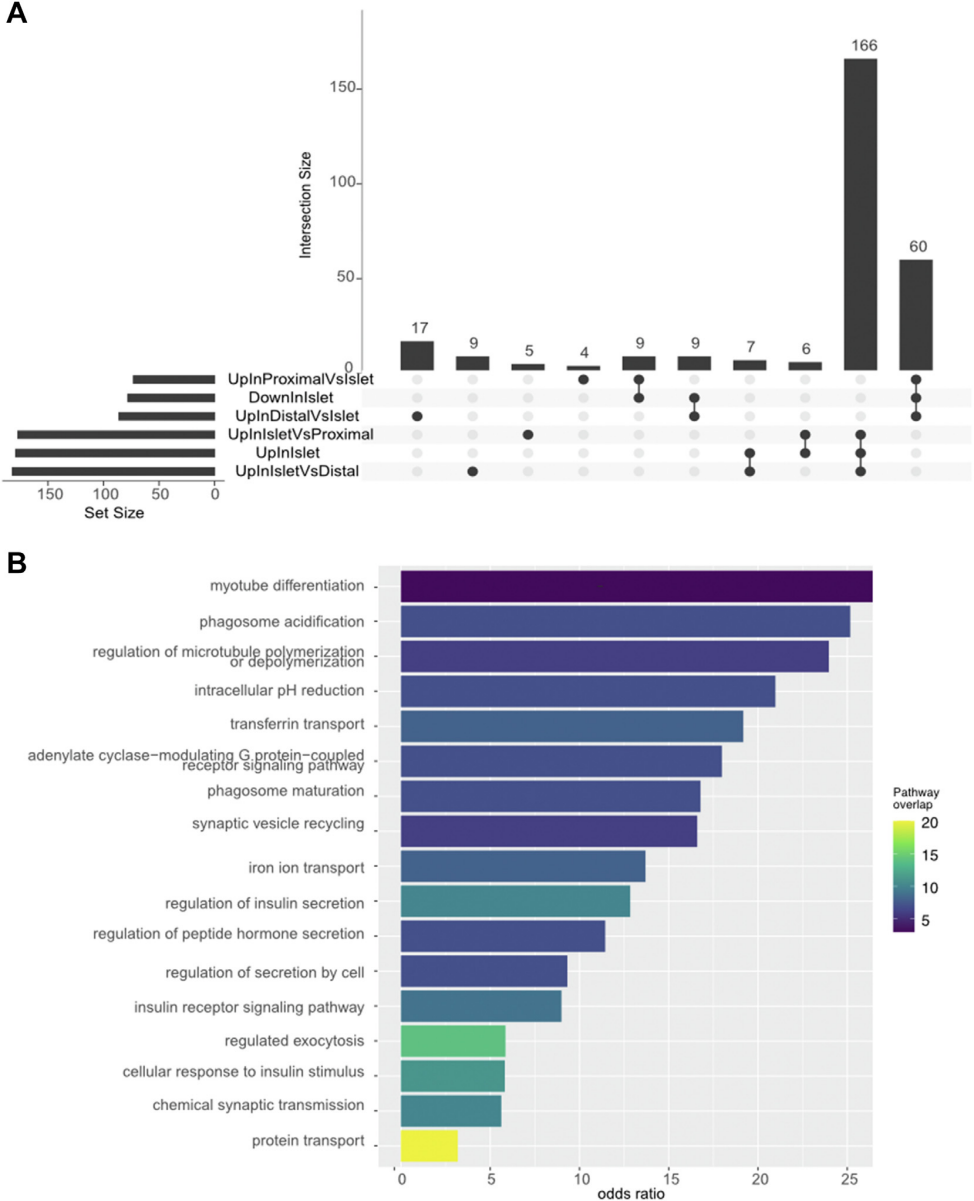
**Pooled differential expression between discrete image regions.***A*, the number of differentially (corrected *p* < 0.05) expressed proteins identified when comparing different regions. There were no significant protein differences between proximal and distal regions. *B*, the GO biological processes that were enriched in regions with Islet cells.

The protein expression signature of islets is substantially different than the neighboring acinar tissue, though we could not capture the statistics for proteins that were changing within each image because only one islet-containing region was measured. As such, we pooled the images by labeling each voxel by its relationship to the islet and then determined the most significant pathways that had either increased or decreased expression in islets compared to the neighboring microenvironment. For each image, we annotated the five voxels immediately surrounding the islet as “proximal” and the remaining three voxels as “distal” (supplemental Fig. S1). We then grouped the expression of all voxels by these three annotations. We calculated proteins that were significantly (corrected *p* < 0.05) differentially expressed between pairs of regions. The results are shown in the upset plot in Figure 4*A*. As expected, we found the majority of differentially expressed proteins between the islet and other cells were also differentially expressed between the islets and the proximal/distal regions when compared independently. Full differential expression results are depicted in supplemental Table S4.

We then evaluated the pathways that were upregulated in islet cells compared to the non-islet cells. The results, shown for Reactome pathways in Figure 4*B*, depict the most statistically significant terms that were upregulated in the islets. Confirming the role of the islet cell in insulin signaling (66), we see regulation of insulin secretion and response to insulin stimulus as two of the most enriched pathways in the islet cells. We also observed enrichment in intracellular pH, something that has been well-studied in islet cells (67), as well as iron ion transport (68). Full pathway enrichment results are in supplemental Table S5.

### Network Analysis Implicates Related Proteins in Key Islet Pathways

**Supplemental Figure S2:**
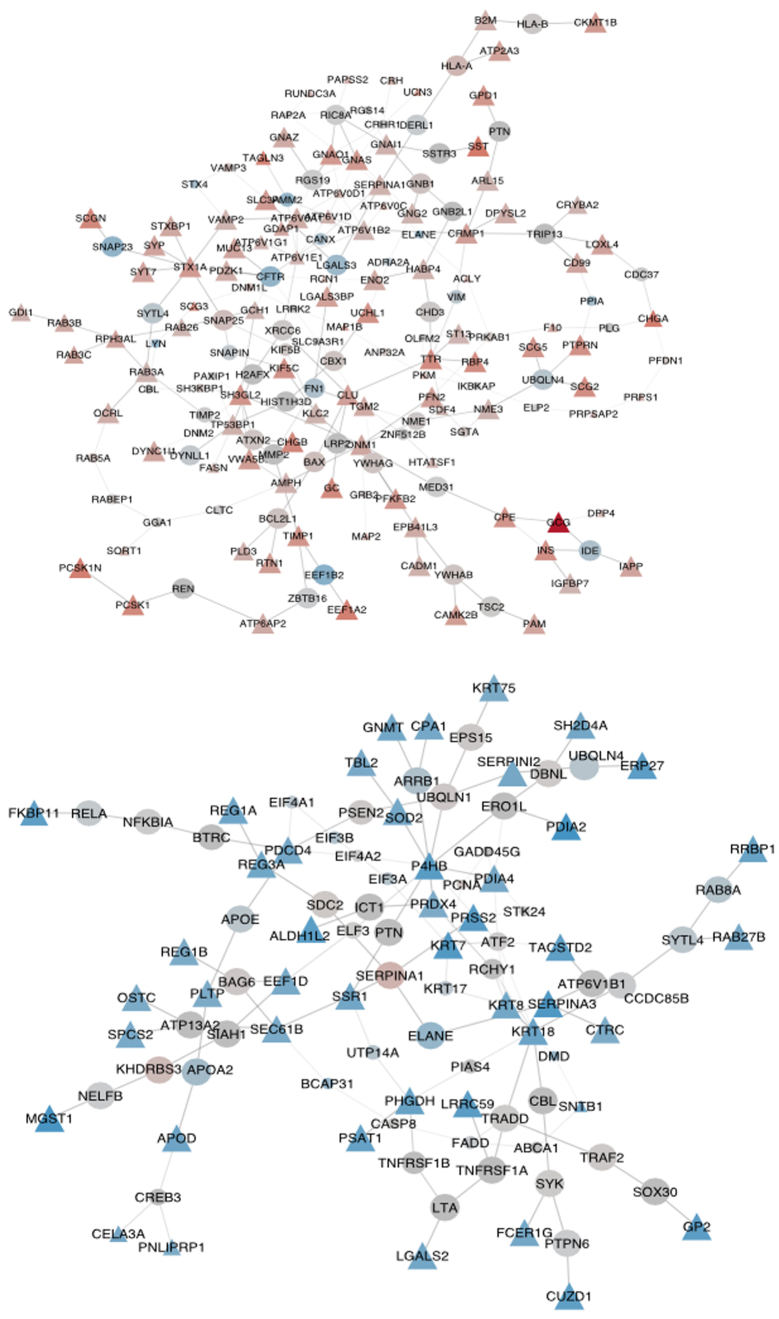
**Complete network results from OmicsIntegrator integration with differentially expressed proteins and STRING protein-protein interaction network.***A*, depicts network derived from proteins up-regulated in islet cells, (*B*) depicts network derived from proteins down-regulated in islet cells. *Triangles* represent differentially expressed proteins, *circles* represent inferred proteins, and shading represents log fold change of proteins between islet regions.

Given the number of proteins derived from the microPOTs measurements (∼6000 proteins per voxel with inferred values for missing data), we explored network inference tools to determine if we could infer biological signaling pathways based on the protein expression alone. Specifically, we used the Prize-collecting Steiner tree algorithm (see Experimental Procedures) to identify the network implicated by proteins upregulated in islets (red, supplemental Fig. S2*A*) and down-regulated in islets (blue, supplemental Fig. S2*B*) by using physical interactions from the STRING database as input (69). As input to the algorithm, we had 261 upregulated proteins and 184 downregulated proteins. The resulting networks were 216 and 179 nodes, respectively, as the algorithm removed proteins that were not connected to others in the interactome and added proteins that maximized the connection of differentially expressed proteins in the network.

**Fig. 5:**
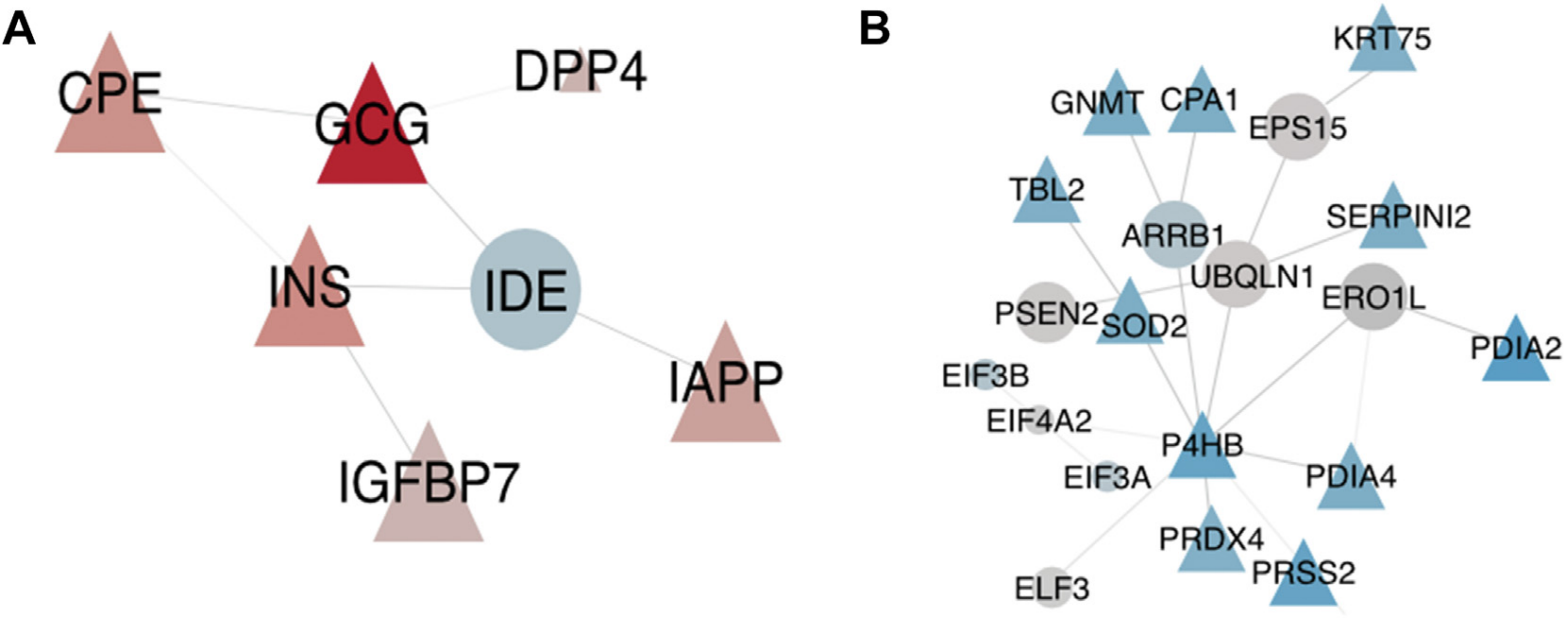
**Islet-specific subnetworks.** Subnetworks resulting from network analysis of proteins that are (A) upregulated in islets compared to those that are (B) downregulated in islets. *Triangles* depict differentially expressed proteins; *circles* indicate those that are integrated through the OmicsIntegrator network analysis. The color of nodes represents the degree of upregulation (*red*) or downregulation (*blue*) in islet cells.

This approach allows us to investigate specific nodes implicated (*i.e.*, not detected experimentally but added *via* the algorithm) in the network (circles in Fig. 5). Specifically, in the islet network, Figure 5*C*, we found IDE, an insulin-degrading enzyme. This protein is implicated in the network as an interactor of many highly expressed proteins including insulin, glucagon, and islet amyloid peptide. Despite being a clear regulator of these proteins (66), insulin-degrading enzyme is somewhat downregulated (but not enough to meet the criteria for statistical significance) in the islets, is essential for the regulation of these proteins and therefore belongs in the signaling network. Similarly, various protein chaperones including P4HB, PDIA4, and ERO1L to be in a cluster in the network that is up-regulated in acinar cells (Fig. 5*D*). This activity underscores the unique importance of protein folding in pancreatic acinar cells (70).

### Distance-Based Metric Reveals Pathway-Level Changes as Distance From Islet Increases

**Fig. 6:**
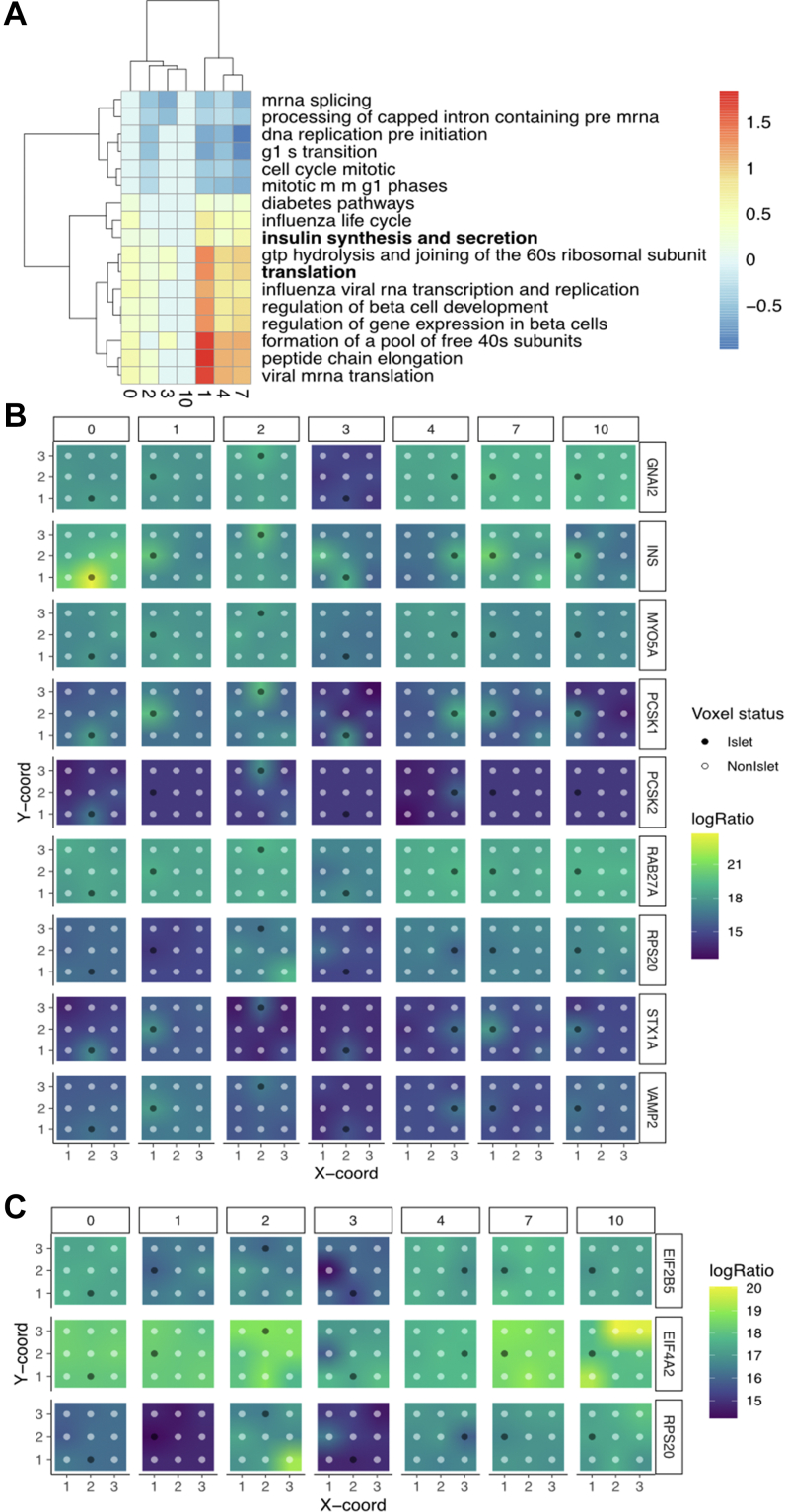
**Distance-based analysis identifies biological pathways and proteins correlated with distance from islet cells.***A*, Z-scores of biological pathways that are significantly (corrected *p* < 0.05) correlated with distance in at least four images. *B*, expression of proteins that are correlated with distance within the Reactome insulin signaling pathway. *C*, expression of proteins that are correlated with distance in Reactome translation pathway.

To explore more subtle changes between the voxels beyond the differential expression analyses highlighted above, we experimented with an alternate approach that searched for signals that permeated through the tissue from the voxel containing the islet. To do this, measured the Spearman rank correlation of the expression of each protein to the distance of the voxel to the islet cell. We then searched for biological pathways using the Reactome pathways that were enriched in proteins with a high positive correlation (more active farther from the islet) or a highly negative correlation (more active closer to the islet). Specifically, we computed the results for each of the seven images, to ensure that we were getting similar results, and plotted the z-scores of the test statistic of those terms that are statistically significant in at least four images in Figure 6*A* with the full results described in supplemental Table S6.

We found numerous pathways, such as insulin synthesis and translation, to be statistically significantly enriched across multiple images. To probe these further, we selected those proteins from the pathway whose correlation test statistic was significant (*p* < 0.05) and plotted those proteins involved in insulin signaling (Fig. 6*B*) and translation (Fig. 6*C*). These both agree with the findings of these biological pathways using standard enrichment analysis with image pooling as well as the network analysis, suggesting that correlation statistics can also be informative in cases where replicates are scarce.

## Discussion

Here we introduce a series of computational tools that show how the microPOTS spatial proteomics platform can be utilized in a clinical setting to characterize specific biological pathways that are uniquely expressed in pancreatic islet cells. We can robustly characterize >6000 proteins in each sample and identify, within each sample, islet-specific biological processes. Our computational analysis enhances initial proteomic resolution through network integration and distance analysis.

These results highlight the distinction between insulin secretion, an exclusive islet cell activity, and insulin signaling, which is clearly enriched in the neighboring acinar cells. Network analysis highlights the role of ER folding proteins that are upregulated in the acinar tissue, in line with the role of these cells in protein folding and secretion. Furthermore, we detect translation-related activity in a gradient that provides a complementary view of the secretory role of acinar cells (70, 71). Clearly, these are hypothesis-generating observations, and substantiation of these hypotheses would require careful mechanistic experiments, possibly using a spatially controlled system such as a pancreas-on-a-chip system. However, by carrying out multiple computational tests and arriving at similar conclusions we can leverage these software approaches together with additional microPOTS experiments. The value of this unbiased spatial proteomic approach is that it suggests targets for genetic manipulation in future experiments.

In summary, we believe the technology and analysis procedures described herein enable a diverse set of applications of proteomics in the clinical setting. There are many diseases, such as cancer or type 1 diabetes, in which a small group of cells can cause a large amount of damage. As such, these technologies are imperative to enable the study of specific signaling activities that enable these cells to affect the neighboring tissue to cause systemic disease. Going forward we plan to collect additional tissue measurements to confirm the results we found in a single pancreas across diverse patients.

## Data Availability

Raw data has been uploaded to massive and is available at https://massive.ucsd.edu/ProteoSAFe/dataset.jsp?task=a49b0c275ed64142b3c158231bb505ef. Processed data is available at http://synapse.org/microPotsPanc. A Synapse account is required to access the data. All code to analyze the data and recreate the analysis is available at http://github.com/pnnl-compbio/hubmap.

## Supplemental data

This article contains supplemental data.

## Conflict of interest

The authors declare no competing interests.

## Figures and Tables

**Supplemental Figure S3:**
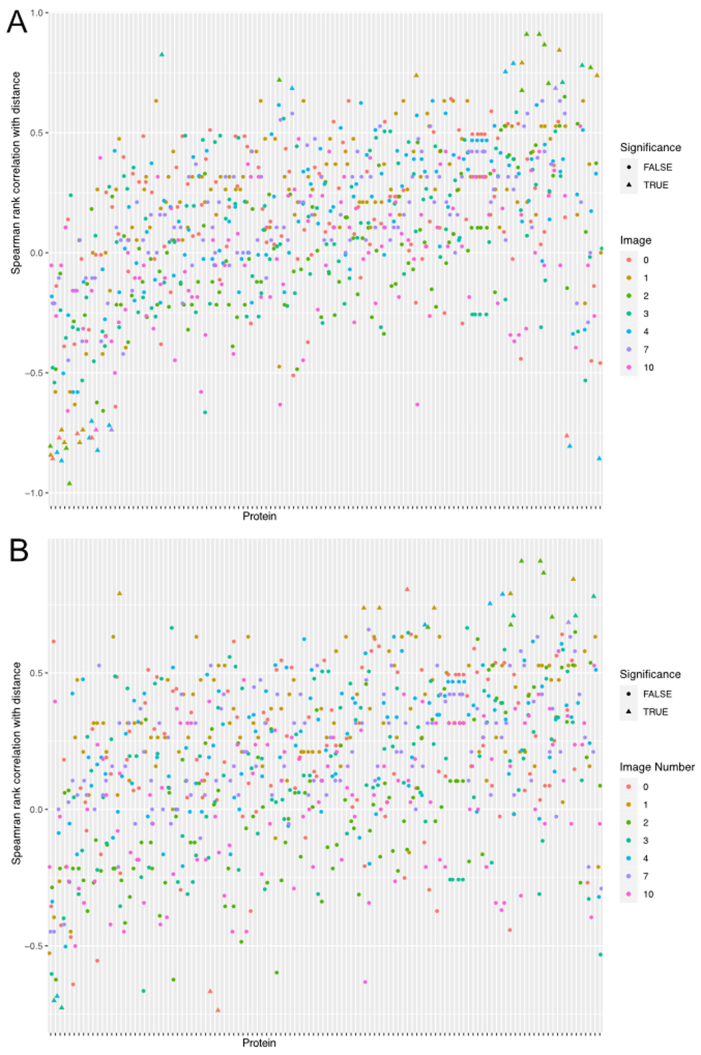
**Individual correlation values of proteins in (A) insulin signaling and (B) translation Reactome pathways.***Color* indicates image in which the correlation was measured, *shape* indicates statistical significance (*p* < 0.05).

## Supplementary Data

Supplemental Table S1 Processed proteomics data table with non-normalized log2 intensities at the protein level. **Supplemental Table S2**Processed proteomics data table with non-normalized log2 intensities at the peptide level. **Supplemental Table S3**List of proteins and their unnormalized log2 ratios across each voxel and image. **Supplemental Table S4**LIMMA results of all differential expression comparisons evaluated in Figure 3. **Supplemental Table S5**Pathway enrichment using leapR comparing islets to non-islet regions and GO biological pathways. **Supplemental Table S6**Individual correlation values for all proteins in each image.

## References

[1] Weir G.C., Bonner-Weir S. (2013). Islet β cell mass in diabetes and how it relates to function, birth, and death. Ann. N. Y Acad. Sci..

[2] Gepts W., Lecompte P.M. (1981). The pancreatic islets in diabetes. Am. J. Med..

[3] Mokdad A.H., Serdula M.K., Dietz W.H., Bowman B.A., Marks J.S., Koplan J.P. (2000). The continuing epidemic of obesity in the United States. JAMA.

[4] Zhu Y., Clair G., Chrisler W.B., Shen Y., Zhao R., Shukla A.K. (2018). Proteomic analysis of single mammalian cells enabled by microfluidic nanodroplet sample preparation and ultrasensitive NanoLC-MS. Angew. Chem. Int. Ed Engl..

[5] Zhu Y., Piehowski P.D., Zhao R., Chen J., Shen Y., Moore R.J. (2018). Nanodroplet processing platform for deep and quantitative proteome profiling of 10-100 mammalian cells. Nat. Commun..

[6] Swensen A.C., Veličković D., Williams S.M., Moore R.J., Day L.Z., Niessen S. (2022). Proteomic profiling of intra-islet features reveals substructure-specific protein signatures. Mol. Cell. Proteomics.

[7] Pontén F., Schwenk J.M., Asplund A., Edqvist P.H.D. (2011). The human protein atlas as a proteomic resource for biomarker discovery. J. Intern. Med..

[8] Kim M.S., Pinto S.M., Getnet D., Nirujogi R.S., Manda S.S., Chaerkady R. (2014). A draft map of the human proteome. Nature.

[9] Tiscornia O.M., Negri G.A., Otero G., López Mingorance F.N., Waisman H., Tiscornia-Wasserman P.G. (2015). Pancreatic polypeptide: a review of its involvement in neuro-endocrine reflexes, islet-acinar interactions and ethanol-evoked physiopatologic pancreatic gland changes. Acta Gastroenterol. Latinoam..

[10] Pierzynowski S.G., Gregory P.C., Filip R., Woliński J., Pierzynowska K.G. (2018). Glucose homeostasis dependency on acini-islet-acinar (AIA) axis communication: a new possible pathophysiological hypothesis regarding diabetes mellitus. Nutr. Diabetes.

[11] Nakagawa A., Stagner J.I., Samols E. (1995). *In situ* binding of islet hormones in the isolated perfused rat pancreas: evidence for local high concentrations of islet hormones via the islet-acinar axis. Diabetologia.

[12] Barreto S.G., Carati C.J., Toouli J., Saccone G.T.P. (2010). The islet-acinar axis of the pancreas: more than just insulin. Am. J. Physiol. Gastrointest. Liver Physiol..

[13] Campbell-Thompson M., Rodriguez-Calvo T., Battaglia M. (2015). Abnormalities of the exocrine pancreas in type 1 Diabetes. Curr. Diab. Rep..

[14] Campbell-Thompson M.L., Filipp S.L., Grajo J.R., Nambam B., Beegle R., Middlebrooks E.H. (2019). Relative pancreas volume is reduced in first-degree relatives of patients with type 1 Diabetes. Diabetes Care.

[15] Ross J.J., Wasserfall C.H., Bacher R., Perry D.J., McGrail K., Posgai A.L. (2021). Exocrine pancreatic enzymes are a serological biomarker for type 1 diabetes staging and pancreas size. Diabetes.

[16] Regev A., Teichmann S.A., Lander E.S., Amit I., Benoist C., Birney E. (2017). The human cell atlas. Elife.

[17] Snyder M.P., Lin S., Posgai A., Atkinson M., Regev A., Rood J. (2019). The human body at cellular resolution: the NIH human biomolecular atlas program. Nature.

[18] Wang J., Ma Z., Carr S.A., Mertins P., Zhang H., Zhang Z. (2017). Proteome profiling outperforms transcriptome profiling for coexpression based gene function prediction. Mol. Cell. Proteomics.

[19] Duraiyan J., Govindarajan R., Kaliyappan K., Palanisamy M. (2012). Applications of immunohistochemistry. J. Pharm. Bioallied Sci..

[20] Bodenmiller B. (2016). Multiplexed epitope-based tissue imaging for discovery and healthcare applications. Cell Syst..

[21] Black S., Phillips D., Hickey J.W., Kennedy-Darling J., Venkataraaman V.G., Samusik N. (2021). CODEX multiplexed tissue imaging with DNA-conjugated antibodies. Nat. Protoc..

[22] Goltsev Y., Samusik N., Kennedy-Darling J., Bhate S., Hale M., Vazquez G. (2018). Deep profiling of mouse splenic architecture with CODEX multiplexed imaging. Cell.

[23] Su P., McGee J.P., Durbin K.R., Hollas M.A.R., Yang M., Neumann E.K. (2022). Highly multiplexed, label-free proteoform imaging of tissues by individual ion mass spectrometry. Sci. Adv..

[24] Hsu C.C., Chou P.T., Zare R.N. (2015). Imaging of proteins in tissue samples using nanospray desorption electrospray ionization mass spectrometry. Anal. Chem..

[25] Ryan D.J., Spraggins J.M., Caprioli R.M. (2019). Protein identification strategies in MALDI imaging mass spectrometry: a brief review. Curr. Opin. Chem. Biol..

[26] van der Veeken J., Zhong Y., Sharma R., Mazutis L., Dao P., Pe’er D. (2019). Natural genetic variation reveals key features of epigenetic and transcriptional memory in virus-specific CD8 T Cells. Immunity.

[27] Vaysse P.M., Heeren R.M.A., Porta T., Balluff B. (2017). Mass spectrometry imaging for clinical research – latest developments, applications, and current limitations. Analyst.

[28] Mao Y., Wang X., Huang P., Tian R. (2021). Spatial proteomics for understanding the tissue microenvironment. Analyst.

[29] Griesser E., Wyatt H., Ten Have S., Stierstorfer B., Lenter M., Lamond A.I. (2020). Quantitative profiling of the human substantia nigra proteome from laser-capture microdissected FFPE tissue. Mol. Cell. Proteomics.

[30] Chen W., Wang S., Adhikari S., Deng Z., Wang L., Chen L. (2016). Simple and integrated Spintip-based technology applied for deep proteome profiling. Anal. Chem..

[31] Davis S., Scott C., Ansorge O., Fischer R. (2019). Development of a sensitive, scalable method for spatial, cell-type-resolved proteomics of the human brain. J. Proteome Res..

[32] Clair G., Piehowski P.D., Nicola T., Kitzmiller J.A., Huang E.L., Zink E.M. (2016). Spatially-resolved proteomics: rapid quantitative analysis of laser capture microdissected alveolar tissue samples. Sci. Rep..

[33] Ryan D.J., Patterson N.H., Putnam N.E., Wilde A.D., Weiss A., Perry W.J. (2019). MicroLESA: integrating autofluorescence microscopy, in situ micro-digestions, and liquid extraction surface analysis for high spatial resolution targeted proteomic studies. Anal. Chem..

[34] Wiśniewski J.R., Ostasiewicz P., Mann M. (2011). High recovery FASP applied to the proteomic analysis of microdissected formalin fixed paraffin embedded cancer tissues retrieves known colon cancer markers. J. Proteome Res..

[35] Wiśniewski J.R., Ostasiewicz P., Duś K., Zielińska D.F., Gnad F., Mann M. (2012). Extensive quantitative remodeling of the proteome between normal colon tissue and adenocarcinoma. Mol. Syst. Biol..

[36] Saha-Shah A., Esmaeili M., Sidoli S., Hwang H., Yang J., Klein P.S. (2019). Single cell proteomics by data-independent acquisition to study embryonic asymmetry in Xenopus laevis. Anal. Chem..

[37] Choi S.B., Polter A.M., Nemes P. (2022). Patch-clamp proteomics of single neurons in tissue using electrophysiology and subcellular capillary electrophoresis mass spectrometry. Anal. Chem..

[38] Johnson K.R., Greguš M., Kostas J.C., Ivanov A.R. (2022). Capillary electrophoresis coupled to electrospray ionization tandem mass spectrometry for ultra-sensitive proteomic analysis of limited samples. Anal. Chem..

[39] Choi S.B., Lombard-Banek C., Muñoz-LLancao P., Manzini M.C., Nemes P. (2018). Enhanced peptide detection toward single-neuron proteomics by reversed-phase fractionation capillary electrophoresis mass spectrometry. J. Am. Soc. Mass Spectrom..

[40] Shen X., Yang Z., McCool E.N., Lubeckyj R.A., Chen D., Sun L. (2019). Capillary zone electrophoresis-mass spectrometry for top-down proteomics. Trends Analyt. Chem..

[41] Zhang Z., Dovichi N.J. (2018). Optimization of mass spectrometric parameters improve the identification performance of capillary zone electrophoresis for single-shot bottom-up proteomics analysis. Anal. Chim. Acta.

[42] Kwon Y., Piehowski P.D., Zhao R., Sontag R.L., Moore R.J., Burnum-Johnson K.E. (2022). Hanging drop sample preparation improves sensitivity of spatial proteomics. Lab Chip.

[43] Zhu Y., Dou M., Piehowski P.D., Liang Y., Wang F., Chu R.K. (2018). Spatially resolved proteome mapping of laser capture microdissected tissue with automated sample transfer to nanodroplets. Mol. Cell. Proteomics.

[44] Piehowski P.D., Zhu Y., Bramer L.M., Stratton K.G., Zhao R., Orton D.J. (2020). Automated mass spectrometry imaging of over 2000 proteins from tissue sections at 100-μm spatial resolution. Nat. Commun..

[45] Dou M., Tsai C.F., Piehowski P.D., Wang Y., Fillmore T.L., Zhao R. (2019). Automated nanoflow two-dimensional reversed-phase liquid chromatography system enables in-depth proteome and phosphoproteome profiling of nanoscale samples. Anal. Chem..

[46] Weke K., Singh A., Uwugiaren N., Alfaro J.A., Wang T., Hupp T.R. (2021). MicroPOTS Analysis of Barrett’s Esophageal cell line models identifies proteomic changes after physiologic and radiation stress. J. Proteome Res..

[47] Xu K., Liang Y., Piehowski P.D., Dou M., Schwarz K.C., Zhao R. (2019). Benchtop-compatible sample processing workflow for proteome profiling of < 100 mammalian cells. Anal. Bioanal. Chem..

[48] Budnik B., Levy E., Harmange G., Slavov N. (2018). SCoPE-MS: mass spectrometry of single mammalian cells quantifies proteome heterogeneity during cell differentiation. Genome Biol..

[49] Cheung T.K., Lee C.Y., Bayer F.P., McCoy A., Kuster B., Rose C.M. (2021). Defining the carrier proteome limit for single-cell proteomics. Nat. Methods.

[50] Fedorec A.J.H., Robinson C.M., Wen K.Y., Barnes C.P. (2020). FlopR: an open source software package for calibration and normalization of plate reader and flow cytometry data. ACS Synth. Biol..

[51] Finak G., Frelinger J., Jiang W., Newell E.W., Ramey J., Davis M.M. (2014). OpenCyto: an open source infrastructure for scalable, robust, reproducible, and automated, end-to-end flow cytometry data analysis. PLoS Comput. Biol..

[52] Burton R.J., Ahmed R., Cuff S.M., Baker S., Artemiou A., Eberl M. (2021). CytoPy: an autonomous cytometry analysis framework. PLoS Comput. Biol..

[53] Righelli D., Weber L.M., Crowell H.L., Pardo B., Collado-Torres L., Ghazanfar S. (2022). SpatialExperiment: infrastructure for spatially resolved transcriptomics data in R using Bioconductor. Bioinformatics.

[54] Pardo B., Spangler A., Weber L.M., Page S.C., Hicks S.C., Jaffe A.E. (2022). spatialLIBD: an R/Bioconductor package to visualize spatially-resolved transcriptomics data. BMC Genomics.

[55] Zhao E., Stone M.R., Ren X., Guenthoer J., Smythe K.S., Pulliam T. (2021). Spatial transcriptomics at subspot resolution with BayesSpace. Nat. Biotechnol..

[56] Solimando A.G., Desantis V., Da Vià M.C. (2023). Visualizing the interactions shaping the imaging of the microenvironment in human cancers. Methods Mol. Biol..

[57] Veličković M., Fillmore T.L., Attah K., Posso C., Pino J.C., Zhao R. (2023). Coupling microdroplet-based sample preparation, multiplexed isobaric labeling, and nanoflow peptide fractionation for deep proteome profiling of tissue microenvironment. bioRxiv.

[58] Gibbons B.C., Chambers M.C., Monroe M.E., Tabb D.L., Payne S.H. (2015). Correcting systematic bias and instrument measurement drift with mzRefinery. Bioinformatics.

[59] Kim S., Pevzner P.A. (2014). Universal database search tool for proteomics. Nat. Commun..

[60] Kim S., Gupta N., Pevzner P.A. (2008). Spectral probabilities and generating functions of tandem mass spectra: a strike against decoy databases. J. Proteome Res..

[61] Monroe M.E., Shaw J.L., Daly D.S., Adkins J.N., Smith R.D. (2008). MASIC: a software program for fast quantitation and flexible visualization of chromatographic profiles from detected LC-MS(/MS) features. Comput. Biol. Chem..

[62] Conway J.R., Lex A., Gehlenborg N. (2017). UpSetR: an R package for the visualization of intersecting sets and their properties. Bioinformatics.

[63] Danna V., Mitchell H., Anderson L., Godinez I., Gosline S.J.C., Teeguarden J. (2021). leapR: an R Package for multiomic pathway analysis. J. Proteome Res..

[64] Akhmedov M., Kedaigle A., Chong R.E., Montemanni R., Bertoni F., Fraenkel E. (2017). PCSF: an R-package for network-based interpretation of high-throughput data. PLoS Comput. Biol..

[65] Tuncbag N., Gosline S.J.C., Kedaigle A., Soltis A.R., Gitter A., Fraenkel E. (2016). Network-based interpretation of diverse high-throughput datasets through the omics integrator software package. PLoS Comput. Biol..

[66] Heaton E.S., Jin S. (2022). Importance of multiple endocrine cell types in islet organoids for type 1 diabetes treatment. Transl. Res..

[67] Lindström P., Sehlin J. (1984). Effect of glucose on the intracellular pH of pancreatic islet cells. Biochem. J..

[68] Marku A., Galli A., Marciani P., Dule N., Perego C., Castagna M. (2021). Iron metabolism in pancreatic beta-cell function and dysfunction. Cells.

[69] Szklarczyk D., Gable A.L., Nastou K.C., Lyon D., Kirsch R., Pyysalo S. (2021). The STRING database in 2021: customizable protein–protein networks, and functional characterization of user-uploaded gene/measurement sets. Nucleic Acids Res..

[70] Kubisch C.H., Logsdon C.D. (2008). Endoplasmic reticulum stress and the pancreatic acinar cell. Expert Rev. Gastroenterol. Hepatol..

[71] Sans M.D., Williams J.A. (2002). Translational control of protein synthesis in pancreatic acinar cells. Int. J. Gastrointest. Cancer.

